# The value of metabolic parameters and textural analysis in predicting prognosis in locally advanced cervical cancer treated with chemoradiotherapy

**DOI:** 10.1007/s00066-022-01900-x

**Published:** 2022-01-24

**Authors:** Sara Pedraza, Alexander P. Seiffert, Pilar Sarandeses, Beatriz Muñoz-Lopez, Enrique J. Gómez, Patricia Sánchez-González, José F. Pérez-Regadera

**Affiliations:** 1 Faculty of Medicine, Universidad Complutense de Madrid and Department of Radiation Oncology, Hospital Universitario 12 de Octubre, Avenida de Córdoba, s/n. 28041 Madrid, Spain; 2 Biomedical Engineering and Telemedicine Centre, ETSI Telecomunicación, Center for Biomedical Technology, Universidad Politécnica de Madrid, Madrid, Spain; 3 Department of Nuclear Medicine, Hospital Universitario 12 de Octubre, Madrid, Spain; 4 Centro de Investigación Biomédica en Red de Bioingeniería, Biomateriales y Nanomedicina (CIBER-BBN), Madrid, Spain

**Keywords:** Uterine cervical neoplasms, Chemoradiotherapy, Prognosis, Positron emission tomography–computed tomography

## Abstract

**Objective:**

The aim of the study was to assess the impact of clinical and metabolic parameters derived from ^18^F-FDG PET/CT (positron emission tomography–computed tomography) in patients with locally advanced cervical cancer (LACC) on prognosis.

**Methods:**

Patients with LACC of stage IB2-IVA treated by primary radiochemotherapy followed by brachytherapy were enrolled in this retrospective study. Indexes derived from standardized uptake value (SUV), metabolic tumor volume (MTV), total lesion glycolysis (TLG), and textural features of the primary tumor were measured for each patient. Overall survival (OS) and recurrence-free survival (RFS) rates were calculated according to Kaplan–Meier and survival curves were compared using the log-rank test. Uni- and multivariate analyses were performed using the Cox regression model.

**Results:**

A total of 116 patients were included. Median follow-up was 58 months (range: 1–129). A total of 36 (31%) patients died. Five-year OS and RFS rates were 69 and 60%, respectively. Univariate analyses indicated that FIGO stage, the presence of hydronephrosis, high CYFRA 21.1 levels, and textural features had a significant impact on OS and RFS. MTV as well as SCC-Ag concentration were also significantly associated with OS. On multivariate analysis, the presence of hydronephrosis, CYFRA 21.1, and sphericity were independent prognostics factors for OS and RFS. Also, SCC-Ag level, MTV, and GLZLM (gray-level zone length matrix) ZLNU (zone length non-uniformity) were significantly associated with OS.

**Conclusion:**

Classical prognostic factors and tumor heterogeneity on pretreatment PET/CT were significantly associated with prognosis in patients with LACC.

## Introduction

Cervical cancer (CC) is one of the most common types of cancer in women worldwide, with over 500,000 newly diagnosed cases and over 300,000 deaths per year [1]. Almost half of all patients are diagnosed with a locally advanced disease stage [2], which according to the International Federation of Gynecology and Obstetrics (FIGO) system includes IB2-IVA stages [3]. In this group, combined chemoradiotherapy (CRT) based on cisplatin has been the standard treatment since 1999 [4–6]. Nevertheless, in spite of a high-complexity treatment, global survival at 5 years is estimated at around 65% [7] and one third of patients relapse within the first 2 years from treatment [8].

With the technological development that has occurred in recent decades in the field of radiation oncology, better disease control is currently reported compared to historical cohorts [9, 10]. Because intensity-modulated radiotherapy (RT) treatment planning can optimize volume target while sparing organs [5] and high-precision image-guided adaptative brachytherapy allows dose escalation to tumor [9], individualizing treatment according to patient characteristics should be promoted [4, 5]. Some clinical and pathological factors such as histological tumor type, tumor size and grade, FIGO stage, lymph node metastasis, lymphovascular invasion, parametrial infiltration, or hydronephrosis are well-stablished prognostic factors [4, 5, 11]. However, cancer disease is heterogeneous and new prognostic factors should be investigated. In this sense, medical imaging plays a key role in guiding treatment decisions.

Positron emission tomography–computed tomography (PET/CT) with ^18^F‑fluorodeoxyglucose (^18^F‑FDG) is used in staging, treatment planning, and response assessment in locally advanced cervical cancer (LACC) [3, 12, 13]. ^18^F‑FDG PET/CT also has a prognostic value and has been used to study recurrence and survival outcome [14–17]. Metabolic parameters based on the standardized uptake value (SUV), like the highest SUV (SUVmax) of the lesion, and the metabolic tumor volume (MTV) as well as total lesion glycolysis (TLG) have been studied previously. These parameters were revealed to be useful in characterizing the tumor and be significant predictors of relapse-free (RFS) and overall survival (OS) [18–24]. Since previous studies include small and heterogeneous samples with a short follow-up, results remain inconclusive.

In addition to metabolic parameters, texture analysis of ^18^F‑FDG PET/CT, also called radiomics, has been used to describe intratumoral heterogeneity and study its relationship to clinical and pathological characteristics in CC [25–29]. In ^18^F‑FDG PET, texture features quantitatively describe the spatial distribution of metabolic activity [30]. These radiomic features could be a useful tool to describe tumor aggressiveness and to predict treatment outcome in cancer patients [30].

In this paper, we investigated the prognostic value of ^18^F‑FDG PET in a study group of 116 patients with LACC. Metabolic parameters were extracted, and textural analysis of the primary tumor was performed to assess the intratumoral heterogeneity. The aim was to study the use of ^18^F‑FDG PET imaging in predicting treatment outcome and survival. Moreover, we aimed to evaluate the extracted ^18^F‑FDG PET measures, especially texture features, in LACC by investigating their relationship to clinical and pathological characteristics and their potential to predict treatment response.

## Materials and methods

### Patients

**Fig. 1:**
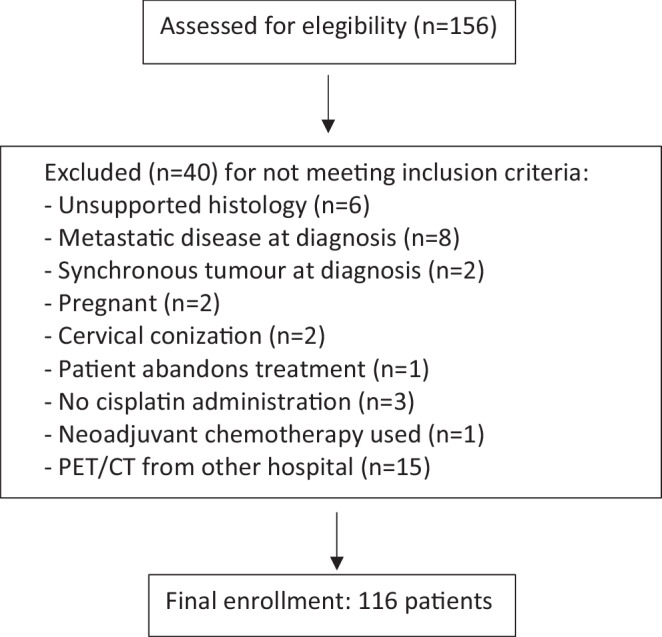
Flowchart of patient selection. *PET/CT* positron emission tomography–computed tomography

From January 2009 to August 2017, 116 consecutive patients with newly diagnosed LACC and treated at 12 de Octubre University Hospital (Madrid, Spain) were enrolled in this retrospective study. All subjects underwent pretreatment ^18^F‑FDG PET/CT for staging and RT planning at our institution and a second one 3 months after completing treatment. Also, clinical exploration and magnetic resonance (MRI) were undergone for staging. Inclusion criteria were as follow: 1) biopsy-confirmed squamous, adenosquamous, or adenocarcinoma histology; 2) IB2-IVA stages according to FIGO staging system [31]; 3) minimum size for primary tumors ≥ 1 cm on MRI; 4) patient fit for radical conservative treatment. Patients under 18 years of age; pregnant women; carriers of the human immunodeficiency virus; patients with synchronous tumor, prior RT, cytostatic other than cisplatin, or neoadjuvant chemotherapy (ChT) were excluded (Fig. 1). Only primary tumors were considered for textural analysis. As lymph nodes are usually small with a low number of voxels involved, quantification mistakes can occur when performing textural analysis and these were therefore not analyzed. Well-established clinical and pathological parameters were reviewed, including age, histology, FIGO stage, primary tumor size, parametrium or hydronephrosis affection, presence of lymph node metastasis, serum squamous cell carcinoma antigen level (SCC-Ag), CYFRA 21.1 level, and Eastern Cooperative Oncology Group (ECOG) status (Table 1). The study was approved by the Ethics Committee for Clinical Research (CEIC No.:18/169) and was carried out in accordance with the ethical principles of the Helsinki Declaration and the Basic Law of Data Protection (Data Protection Act 15/1999).

**Table 1:** Patients and tumoral characteristics

Characteristic	Value (%)
*Age (years), median (range)*	49 (25–84)
*Histology*	
Squamous cell carcinoma	91 (78)
Adenocarcinoma	23 (20)
Adenosquamous cell carcinoma	2 (2)
*FIGO stage*	
IB2	1 (1)
IB3	10 (9)
IIA1	1 (1)
IIB	21 (18)
IIIB	8 (7)
IIIC1	53 (46)
IIIC2	19 (16)
IVA	3 (2)
*Tumor diameter, median (range)*	
< 4 cm:	9 (8)
≥ 4–< 6 cm:	61 (52)
≥ 6 cm:	46 (40)
*Parametrium invasion*	88 (76)
*Hydronephrosis*	10 (9)
*Lymph node affection*	
Pelvis affection	51 (44)
Pelvis + paraaortic affection	9 (8)
*ECOG status*	
ECOG 0–1	98 (84)
ECOG 2–4	18 (16)
*SCC-Ag (ng/ml), median (range)*	4.2 (0.5–221)
*CYFRA 21.1 (ng/ml), median (range)*	3.025 (0.23–246)

Values are presented as mean, range, or number and percentage.

*FIGO* Federation of Gynecology and Obstetrics, *ECOG* Eastern Cooperative Oncology Group, *SCC-Ag* serum squamous cell carcinoma antigen, *CYFRA 21.1* serum cytokeratin fragment 21.1

### Treatment

All patients received a combination of radical external beam RT (EBRT) and brachytherapy (BT). The ^18^F‑FDG PET/CT imaging was used for radiation treatment planning. EBRT was delivered to the whole pelvis with customized shielding, to a dose ranging between 45 and 46 Gy (1.8–2 Gy/fraction, Monday through Friday) using high-energy photons (18 or 20 MV) from a linear accelerator (Siemens Primus®, Siemens AG, Erlangen, Germany; or Clinac IX®, Varian Medical Systems, Palo Alto, CA, USA). For patients with paraaortic nodal involvement, bulky tumor, or iliac pelvic node metastasis, RT extended to the paraaortic zone was planned, prescribing 45 Gy (1.8 Gy/fraction). An additional 10-Gy boost was indicated in case of lymph node affection ≥ 1 cm or parametrial affection. Conformal radiotherapy was performed in nearly all cases, except in 7 patients who received volumetric modulated arc therapy (VMAT). Concurrent weekly cisplatin (40 mg/m^2^) was administered in all patients (median cycles: 5, range: 2–7) to a maximal dose of 70 mg. After finishing external beam radiotherapy and cisplatin, high-dose-rate intracavitary brachytherapy was performed. CT was used for planning until 2011, whereafter MRI was used. A personalized vaginal mold was used in all patients and 21–28 Gy in 3–4 weekly fractions were prescribed to the CTV (clinical tumor volume).

### ^18^F-FDG PET/CT image acquisition

All patients were scanned using a Biograph PET/CT scanner with three-dimensional reconstruction of PET and a six-slice helical CT system (Siemens Medical Solutions, Malver, PA, USA) under the same institutional protocol. Patients were instructed to fast for at least 6 h before undergoing ^18^F‑FDG PET/CT, which was conducted approximately 60 min after the administration of 4–5 MBq/kg of ^18^F‑FDG. Oral contrast and intravenous contrast, if no allergies were reported, were applied in all cases. Bladder catheterization before injection of ^18^F‑FDG was used to minimized urinary tract activity. First, CT was performed from head to proximal thighs (tube voltage: 130 Kv, tube current: 60 mA, slice thickness: 5 mm) with the patient in the supine position, arms above head, and with customized radiotherapy immobilization systems. Patients were scanned on a carbon-fiber table, breathing shallowly, and without repositioning the patient on the table when PET was performed. CT images were used for attenuation correction of PET scans. The PET emission scan was also obtained from the skull base to the thigh at 3 min per bed position in a three-dimensional image. Images were reconstructed with a 168 × 168 matrix using ordered the subset expectation maximization algorithm (21 subsets, 3 interactions).

### Image analysis

The ^18^F‑FDG PET/CT images (CT, PET, and PET/CT axial, coronal, and sagittal) were visually interpreted by a single nuclear medicine physician with more than 10 years of experience, evaluating abnormal ^18^F‑FDG uptake at primary tumor site, lymph nodes, or distant sites. Lymph nodes were considered malignant if ^18^F‑FDG uptake was greater than the background tissue or blood pool activity. For subcentimeter but suspicious malignant lymph nodes, other pathological features such as shape and central necrosis were taken into consideration. In case of doubt, consensus among another nuclear physicians was obtained. Primary tumor identification and delineation were performed manually on PET images. Special caution was taken in case of tumor contact with the bladder. A 41% threshold of SUVmax was then applied to objectively distinguish metabolically active tumor tissue from background, segmenting the final tumoral volume of interest (VOI) [32]. Firstly, the following SUV parameters were extracted: SUVmax, SUVmean (the average uptake in the tumor), SUVpeak (local average within a small region centered on the voxel with the highest SUV), SUVmin (minimum uptake in the tumor), SUVstd (standard deviation of SUVs in the lesion), and SUVQ1, SUVQ2, SUVQ3 (quartiles of SUVs in the lesion). Also, MTV, which represents the volume of the primary tumor, and TLG (TLG = MTV × SUVmean) were studied. Secondly, textural features to evaluate the heterogeneity were extracted for each VOI. Depending on the number of voxels that are implied in the matrix, these features were divided into [30]: a) 7 first-order parameters based on the SUV, histogram, and shape; b) 6 second-order texture indices derived from gray-level co-occurrence matrix (GLCM) [33]; c) 25 third- and higher-order texture parameters derived from gray-level zone length matrix (GLZLM) [34], gray-level run length matrix (GLRLM) [35], and neighborhood gray-level difference matrix (NGLDM) [36]. All patients had a double reading of metabolic parameters performed by another physician to check the accuracy of the results. LIFEx software (Local Image Feature Extraction, www.lifexsoft.org) was used for the entire feature extraction process [37].

### Outcome evaluation

Our protocol of surveillance consisted of physical and gynecological examination and laboratory tests (complete blood count, kidney and hepatic function, SCC-Ag level) every 3 months for 2 years, every 6 months for the next 3 years, and then annually. Three months after finishing treatment, pelvic MRI and ^18^F‑FDG PET/CT are performed in all patients to assess response. Every 6 months, pelvic MRI, abdominopelvic CT, and chest radiography are obtained for 5 years and once per year thereafter. ^18^F‑FDG PET/CT is performed in case of suspected recurrence. Pelvic recurrences, metastatic disease, and deaths were recorded.

### Statistical analysis

Continuous data were presented as median and range and categorical data were expressed as frequencies and percentages. Time-to-event was calculated from the last day of RT. The event for OS was death (all causes) and the event for recurrence-free survival (RFS) was recurrence (all types).

Univariate survival curves were calculated using the Kaplan–Meier method and differences were evaluated with the log-rank test. Continuous variables were grouped using the median. Optimal cut-offs were not calculated due to the retrospective nature of the study and the lack of a validation cohort [38]. Univariate and multivariate analyses were studied using Cox regression analysis, estimating hazard ratios (HR) with a confidence interval (CI) of 95%. Tumor markers and continuous variables were not stratified in Cox regression analysis to minimize a loss of information [39]. Statistically significant variables in univariate analyses were considered for multivariate analysis and a backward conditional method was used. All data were statistically analyzed on SPSS software version 19.00 (IBM Corp., Armonk, NY, USA). A *p-*value < 0.05 was considered statistically significant.

## Results

**Fig. 2:**
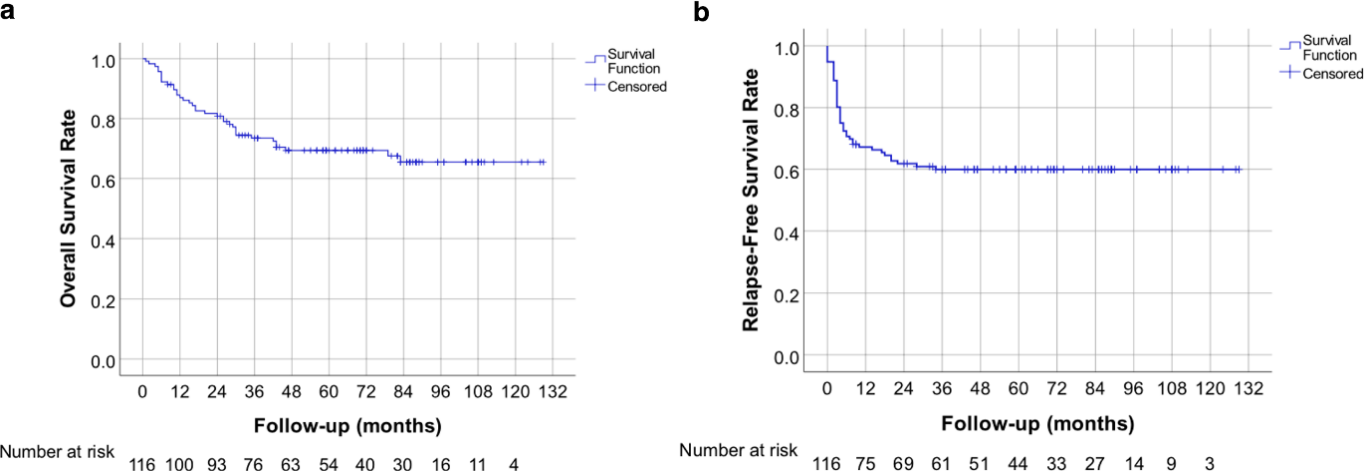
Kaplan–Meier survival curves for overall survival (**a**) and recurrence-free survival (**b**)

The median follow-up time was 58 months (range: 1–129 months); 23 (20%) patients had an inferior follow-up of 24 months (20 patients died and 3 were lost to follow-up). A total of 36/116 (31%) patients died, with 33 cancer-specific deaths and 3 deaths where the patients were tumor free. Relapse occurred in 46 (40%) patients as follows: tumoral progression: 11 (24%); pelvic lymph nodes: 6 (13%); tumoral and pelvic lymph nodes progression: 9 (20%); pelvic relapse and metastasis: 3 (6%); pelvic and paraaortic lymph nodes: 3 (7%); paraaortic lymph nodes: 3 (6%); and distant metastasis: 11 (24%). No intra-treatment progression was verified. The 2‑year, 5‑year, and 10-year OS and RFS rates for all patients were 81, 69, and 65%, and 62, 60, and 60%, respectively. Fig. 2 shows the corresponding Kaplan–Meier survival curves.

The stratified survival rates for OS and RFS are shown in Table 2. Survival was stratified by age, tumor histology, FIGO stage, tumor size, parametrium invasion, hydronephrosis, lymph node affection, ECOG status, SCC-Ag and CYFRA 21.1 levels, and image features. Higher FIGO stage, tumor diameter, the presence of hydronephrosis, as well as higher TLG, MTV, sphericity, compacity, GLRLM gray-level non-uniformity (GLNU), and GLZLM GLNU and zone length non-uniformity (ZLNU), and lower NGLDM coarseness showed significantly lower OS (*p* < 0.05 of log-rank test). On the other hand, tumor histology, the presence of parametrium invasion and hydronephrosis, higher CYFRA 21.1 level, MTV, sphericity, compacity, GLRLM-GLNU, and GLZLM-GLNU and GLZLM-ZLNU showed significantly lower RFS (*p* < 0.05 of log-rank test).

**Table 2:** Overall and recurrence-free survival rates and log-rank tests among patients with locally advanced cervical cancer stratified by clinicopathological and significant image features

Characteristic		Overall survival rate (%)				recurrence-free survival rate (%)			
		2‑year	5‑year	10-year	*P*	2‑year	5‑year	10-year	*P*-value
Age (years)	< 65	80	69	68	0.780	63	61	1	0.781
	≥ 65	78	71	53		56	56	55	
Histology	Squamous cell carcinoma	78	66	61	0.118	58	55	55	0.010*
	Adenocarcinoma	91	86	–		83	83	–	
	Adenosquamous cell carcinoma	100	–	–		00.0	–	–	
FIGO stage	I–II	97	86	86	0.007*	82	78	78	0.009*
	III–IV	74	63	58		54	53	53	
Tumor diameter (cm)	< 4	89	89	–	0.037*	78	78	–	0.115
	≥ 4–< 6	88	74	74		67	65	65	
	≥ 6	70	60	50		52	50	50	
Parametrium invasion	Absent	89	80	–	0.094	78	78	–	0.034*
	Present	78	66	61		57	54	54	
Hydronephrosis	Absent	82	71	70	0.030*	65	64	64	0.005*
	Present	70	47	–		30	20	–	
Lymph node affection	No affection	91	70	65	0.439	66	62	62	0.264
	Pelvis affection	74	70	66		61	61	61	
	Paraaortic affection	–	–	–		–	–	–	
	Pelvis + paraaortic affection	56	56	56		44	44	44	
ECOG status	ECOG 0–1	82	71	66	0.311	64	63	63	0.117
	ECOG 2–4	66	60	60		50	44	44	
SCC-Ag (ng/ml)	< 4.2	91	77	–	0.108	67	67	–	0.087
	≥ 4.2	70	61	61		57	53	53	
CYFRA 21.1 (ng/ml)^a^	< 3.03	86	77	–	0.090	73	73	–	0.005*
	≥ 3.03	75	62	58		51	47	47	
TLG (g)	< 185.48	88	78	–	0.042*	65	65	–	0.193
	≥ 185.48	74	60	57		58	55	55	
MTV (ml)	< 21.12	88	78	74	0.029*	69	69	69	0.036*
	≥ 21.12	74	60	56		55	51	51	
Sphericity	< 1.00	69	60	56	0.012*	50	50	50	0.015*
	≥ 1.00	93	79	75		74	70	70	
Compacity	< 1.51	91	80	–	0.006*	70	70	–	0.021*
	≥ 1.51	70	59	53		53	50	50	
GLRLM-GLNU	< 18.12	90	8	–	0.027*	70	70	–	0.023*
	≥ 18.12	72	61	56		53	50	50	
NGLDM coarseness	< 0.14	70	58	55	0.012*	57	53	53	0.086
	≥ 0.14	91	80	–		67	67	–	
GLZLM GLNU	< 7.68	93	82	–	0.001*	72	72	–	0.006*
	≥ 7.68	69	57	51		51	48	48	
GLZLM ZLNU	< 78.60	91	82	–	0.003*	69	69	–	0.040*
	≥ 78.60	70	56	53		55	51	51	

*FIGO* Federation of Gynecology and Obstetrics, *ECOG* Eastern Cooperative Oncology Group, *SCC-Ag* serum squamous cell carcinoma antigen, *CYFRA 21.1* serum cytokeratin fragment 21.1, *TLG* total lesion glycolysis, *MTV* metabolic tumor volume, *GLRLM* gray-level run length matrix, *GLNU* gray-level non-uniformity, *NGLDM* neighborhood gray-level difference matrix, *GLZLM* gray-level zone length matrix, *ZLNU* zone length non-uniformity

^a^Two cases with missing data were censored

*Significant *p-*value

Univariate Cox regression analysis showed that FIGO stage (HR = 3.736; 95% CI: 1.320–10.571; *p* = 0.013) and hydronephrosis (HR = 2.552; 95% CI: 1.060–6.145; *p* = 0.037), as well as various image features like MTV (HR = 1.009; 95% CI: 1.000–1.018; *p* = 0.048), were significant clinicopathological prognostic characteristics for OS. Multivariate analysis revealed that the presence of hydronephrosis (HR = 3.735; 95% CI: 1.400–9.962; *p* = 0.008), SCC-Ag (HR = 1.015; 95% CI: 1.007–1.022; *p* < 0.001), and CYFRA 21.1 (HR = 1.022; 95% CI: 1.009–1.035; *p* = 0.001) levels, MTV (HR = 0.966; 95% CI: 0.944–0.988; *p* = 0.003), sphericity of the segmented tumor (HR = 0.000; 95% CI: 0.000–0.018; *p* = 0.001), and GLZLM ZLNU (HR = 1.010; 95% CI: 1.004–1.015; *p* = 0.001) were independent predictors of OS. The results are summarized in Table 3.

**Table 3:** Univariate and multivariate Cox-regression analyses for overall survival

Characteristic	Univariate		Multivariate	
	Hazard ratio (95% CI)	*P-*value	Hazard ratio (95% CI)	*P*-value
*Age (years; <* *65 vs. ≥* *65)*	1.133 (0.471–2.722)	0.781	–	–
*FIGO stage (I–II vs. III–IV)*	3.736 (1.320–10.571)	0.013*	–	–
*Tumor diameter (cm)*				
< 4	Ref.	–	–	–
≥ 4–< 6	2.221 (0.293–16.818)	0.440	–	–
≥ 6	4.619 (0.620–34.425)	0.135	–	–
*Parametrium invasion (absent vs. present)*	2.192 (0.852–5.642)	0.104	–	–
*Hydronephrosis (absent vs. present)*	2.552 (1.060–6.145)	0.037*	3.735 (1.400–9.962)	0.008*
*Lymph node affection*				
No affection	Ref.	–	–	–
Pelvis affection	1.121 (0.560–2.244)	0.747	–	–
Pelvis + paraaortic affection	2.017 (0.674–6.039)	0.210	–	–
*ECOG status (0–1 vs. 2–4)*	1.525 (0.667–3.483)	0.317	–	–
*SCC-Ag (ng/ml)*	1.010 (1.003–1.016)	0.002*	1.015 (1.007–1.022)	< 0.001*
*CYFRA 21.1 (ng/ml)*^*a*^	1.022 (1.010–1.035)	< 0.001*	1.022 (1.009–1.035)	0.001*
*MTV (ml)*	1.009 (1.000–1.018)	0.048*	0.966 (0.944–0.988)	0.003*
*Sphericity*	0.001 (0.000–0.118)	0.005*	0.000 (0.000–0.018)	0.001*
*Compacity*	2.371 (1.304–4.312)	0.005*	–	–
*GLCM entropy*	2.278 (1.018–5.096)	0.045*	–	–
*GLRLM RLNU*	1.001 (1.000–1.002)	0.008*	–	–
*GLZLM GLNU*	1.046 (1.013–1.081)	0.006*	–	–
*GLZLM ZLNU*	1.004 (1.002–1.007)	0.001*	1.010 (1.004–1.015)	0.001*

*CI* confidence interval,* FIGO* Federation of Gynecology and Obstetrics, *Ref.* reference, *ECOG* Eastern Cooperative Oncology Group, *SCC-Ag* serum squamous cell carcinoma antigen, *CYFRA 21.1* serum cytokeratin fragment 21.1, *MTV* metabolic tumor volume, *GLCM* gray-level co-occurrence matrix, *GLRLM* gray-level run length matrix, *RLNU* run length non-uniformity, *GLNU* gray-level non-uniformity, *GLZLM* gray-level zone length matrix, *ZLNU* zone length non-uniformity

^a^Two cases with missing data were censored

*Significant *p*-value

Table 4 shows the results of Cox regression analyses for RFS. FIGO stage (HR = 2.722; 95% CI: 1.216–6.092; *p* = 0.015), parametrium invasion (HR = 2.412; 95% CI: 1.022–5.692, *p* = 0.044), hydronephrosis (HR = 2.809; 95% CI: 1.308–6.032; *p* = 0.008), and CYFRA 21.1 level (HR = 2.367; 95% CI: 1.272–4.405; *p* = 0.007) were statistically significant prognostic factors in univariate analysis. The multivariate analysis showed that the presence of hydronephrosis (HR = 3.368; 95% CI: 1.538–7.377; *p* = 0.002), the CYFRA 21.1 level (HR = 1.016; 95% CI: 1.008–1.024; *p* < 0.001), and the sphericity of the segmented tumor (HR = 0.001; 95% CI: 0.000–0.092; *p* = 0.003) were independent predictors of RFS.

**Table 4:** Univariate and multivariate Cox regression analyses for recurrence-free survival

Characteristic	Univariate		Multivariate	
	Hazard ratio (95% CI)	*P*-value	Hazard ratio (95% CI)	*P*-value
*Age (years; <* *65 vs. ≥* *65)*	1.112 (0.518–2.383)	0.786	–	–
*FIGO stage (I–II vs. III–IV)*	2.722 (1.216–6.092)	0.015*	**–**	**–**
*Tumor diameter (cm)*				
< 4	Ref	–	–	–
≥ 4–< 6	1.674 (0.392–7.144)	0.486	–	–
≥ 6	2.780 (0.655–11.801)	0.166	–	–
*Parametrium invasion (absent vs. present)*	2.412 (1.022–5.692)	0.044*	**–**	**–**
*Hydronephrosis (absent vs. present)*	2.809 (1.308–6.032)	0.008*	3.368 (1.538–7.377)	0.002*
*Lymph node affection*				
No affection	Ref	–	–	–
Pelvis affection	1.118 (0.606–2.063)	0.721	–	–
Pelvis + paraaortic affection	2.172 (0.817–5.774)	0.120	–	–
*ECOG status (0–1 vs. 2–4)*	1.720 (0.852–3.472)	0.130	–	–
*SCC-Ag (ng/ml)*	1.007 (1.002–1.013)	0.012	–	–
*CYFRA 21.1 (ng/ml)*^*a*^	1.014 (1.006–1.022)	0.001*	1.016 (1.008–1.024)	< 0.001*
*TLG (g)*	1.001 (1.000–1.001)	0.037*	–	–
*Sphericity*	0.003 (0.000–0.216)	0.008*	0.001 (0.000–0.092)	0.003*
*Compacity*	2.027 (1.194–3.440)	0.009*	–	–
*GLRL RLNU*	1.001 (1.000–1.001)	0.017*	–	–
*GLZLM GLNU*	1.046 (1.015–1.078)	0.003*	–	–
*GLZLM ZLNU*	1.003 (1.001–1.006)	0.003*	–	–

*FIGO* Federation of Gynecology and Obstetrics, *ECOG* Eastern Cooperative Oncology Group, *SCC-Ag* serum squamous cell carcinoma antigen, *CI* confidence interval, *FIGO* Federation of Gynecology and Obstetrics, *Ref.* reference, *ECOG* Eastern Cooperative Oncology Group, *SCC-Ag* serum squamous cell carcinoma antigen, *CYFRA 21.1* serum cytokeratin fragment 21.1, *TLG* total lesion glycolysis, *GLRLM* gray-level run length matrix, *RLNU* run length non-uniformity, *GLNU* gray-level non-uniformity, *GLZLM* gray-level zone length matrix, *ZLNU* zone length non-uniformity

^a^Two cases with missing data were censored

*Significant *p-*value

## Discussion

The objective of the present study was to correlate metabolic parameters and texture analysis from staging ^18^F‑FDG PET/CT in patients with LACC treated with definitive CRT with prognosis by assessing their correlation with OS and RFS. In our study, SUVmax of the primary cervical tumor was not an independent prognostic factor in multivariate analysis but other metabolic features and characteristics were.

Previous researchers have demonstrated prognostic factors related to patient and tumor characteristics in cervical cancer [12, 14, 16, 22–24, 28, 29, 40]. Our study is in line with previous published papers. Nevertheless, having precise prognostic information at diagnosis allows prediction of individual tumor aggressiveness and definition of treatment according to each patient’s characteristics. In this sense, PET/CT offers advantages regarding staging, RT treatment planning, and treatment response assessment in LACC, but questions relating to its role in prognosis remain unclear.

The most studied parameter is SUVmax of the primary cervical tumor. There are two meta-analyses evaluating the prognostic ability of pretreatment PET/CT in cervical cancer. Sarker et al. [22] found that high levels of pretreatment SUVmax in the tumor and pathological lymph nodes of patients treated with surgery and RT or CRT are associated with worse OS and RFS. However, in only two studies did it turn out to be an independent prognostic factor in multivariate analysis. The lack of harmonization of the methods to generate the SUVmax cut-off and therefore the impossibility of generating a universal cut-off value were criticized. In the meta-analysis by Zhao et al. [14], the same trend was observed when analyzing the pretreatment and posttreatment SUVmax values. However, other studies have shown controversial results [16, 23]. In our study, we did not find a significant correlation of primary tumor SUVmax with prognosis.

Other metabolic parameters such as MTV and TLG, as well as texture analysis, may provide a more sensitive measure for the prognosis of cervical cancer by reflecting the entire tumoral volume heterogeneity. It is hypothesized that tumor heterogeneity could be quantified and related to cancer behavior. Han et al.’s [24] meta-analysis demonstrated a higher risk of adverse events or deaths in patients with cervical cancer with high MTV or TLG, in spite of clinical and methodological differences. Pinho et al. [29] studied metabolic parameters in patients with IA2-IVB cervical cancer and showed, using a threshold value of 50% of the SUVmax value, that higher MTV was associated with worse OS (*p* = 0.0005), and lower tumor heterogeneity (calculated by a volume histogram index) with increased OS (*p* = 0.04). Kidd and Grigsby [40] determined intratumoral heterogeneity by the derivative of the volume-threshold function from 40–80%, evidencing a significant association with the risk of pathological lymph nodes (*p* = 0.0207), response to RT (*p* = 0.0017), pelvic recurrence (*p* = 0.0017), and PFS (*p* = 0.03). On the contrary, no significant association was evidenced with textural features in Voglimacci et al.’s [12] investigation, which used a threshold at 40% of SUVmax for MTV delineation. This study includes LACC without paraaortic nodes and only SUVmax had been shown a significant prognostic factor (*p* = 0.0299) [12]. Also, Chen et al. [28] did not include paraaortic metastasis in the investigation and MTV was generated at 50% of SUVmax. The presence of low HGRE was a prognostic factor for low OS (*p* = 0.0001). It should be emphasized that textural analysis of metastatic lymph nodes is technically difficult to achieve due to their small size. In our case, we did not include lymph node textures and VOI was segmented using a threshold value of 41% of SUVmax. A significant association of MTV, sphericity, and GLZLM-ZLNU with OS and of only sphericity with RFS was found.

One strength of our study is the sample size compared to other similar studies. Also, all patients received the same treatment and long-term follow-up, although the lack of a verification group must be considered, and its retrospective nature may induce a selection bias. Some characteristics may affect ^18^F‑FDG PET/CT images, such as differences in histology, tumor volume, or the presence of tumor necrosis. If patients’ characteristics affect the heterogeneity metrics, it could be considered that the differences found in prognosis may be due to this effect. In addition, variations in PET/CT acquisition and reconstruction protocols may also cause differences to other groups and not really be due to real biological discrepancies. Another limitation of the present study is the wide variety of clinical stages with small representation of some of them, which may influence the outcome.

The advantage of maximizing the study of PET/CT images lies in obtaining new tumor characteristics in a noninvasive way without adding costs, allowing analysis of the entire tumor volume even in different timeslots. The interest in metabolic features for radiation oncology is increasing. Radiotherapy is a localized treatment dependent on medical imaging in all its phases. New methods to optimize treatment, improve tumor delineation or help to predict response during treatment will be highly effective. Nevertheless, it is paramount to demonstrate robustness of heterogeneity metric results before assuming their clinical value.

In conclusion, the present study evaluated LACC treated with definitive CRT and showed that intratumoral heterogeneity extracted from pretreatment ^18^F‑FDG PET/CT is the only quantitative parameter that is predictive for OS and RFS. MTV was also a significant prognostic factor for OS. Incorporating intratumoral heterogeneity into biological parameters may help to improve patient stratification to drive therapeutic strategies. To verify the clinical value of metabolic parameters, standard methods for texture as well as multicentric and prospective studies are needed.
